# Editorial: Multiple abiotic stresses: Molecular, physiological, and genetic responses and adaptations in cereals

**DOI:** 10.3389/fpls.2023.1146326

**Published:** 2023-02-21

**Authors:** Nabin Bhusal, Pradeep Sharma, Ranjeet Ranjan Kumar, Sindhu Sareen

**Affiliations:** ^1^ Department of Genetics & Plant Breeding, Agriculture and Forestry University, Bharatpur, Nepal; ^2^ Crop Improvement Division, Indian Institute of Wheat and Barley Research (ICAR), Karnal, India; ^3^ Division of Biochemistry, Indian Agricultural Research Institute (ICAR), New Delhi, India

**Keywords:** abiotic stress, molecular response, physiological response, genetic response, adaptation, cereals

Cereal crops provide approximately 40% of the energy and protein components of the human diet and are vital to the food security of the world (Dunwell, 2014). With the increasing incidence of global warming (expected 1.2–1.9°C higher than ambient during 2021–2040) and extreme weather events, the intensity of various climatic constraints is expected to accelerate, ultimately affecting global cereal crop production (IPCC, 2021). Most of the climatic constraints are abiotic stresses (drought, heat, cold, waterlogging, salinity, mineral deficiency, heavy metal stress, and ultraviolet-B (UV-B), etc.) causing extensive yield losses (Paul et al., 2019; Chaudhry and Sidhu, 2021; Rivero et al., 2022). Estimates have shown that each degree Celsius rise in the global mean temperature may lead to yield losses of 6.0, 3.2, and 7.4% in major cereals, i.e., wheat, rice, and maize, respectively, without effective adaptation and genetic improvement (Zhao et al., 2017).

The occurrence of abiotic stresses singly has less effect than when occurring in combination at different growth stages (Mittler, 2006; Suzuki et al., 2014; Shaar-Moshe et al., 2017). Under field conditions, frequently occurring combined stresses are drought and heat (Mittler, 2006; Lamaoui et al., 2018; Lawas et al., 2018; Nasser et al., 2020), drought and salinity (Paul et al., 2019; Abobatta, 2020), and salinity and waterlogging (Lamaoui et al., 2018; Lawas et al., 2018). The effect of these stresses in combination on crop plants depends on the nature of the interactions between them (Ramu et al., 2016). These multiple stresses induce unique mechanisms (morphophysiological, biochemical, molecular, and genetic) in crop plants for adaptations and cannot be predicted by simply studying each of the different stresses (Suzuki et al., 2014; Zandalinas et al., 2021). Understanding the mechanisms of plant response to multiple stresses is crucial to unravel the complexities of plant responses to stress combinations for the development of climate-resilient crops for future food security (Rivero et al., 2022). Therefore, keeping the above under consideration, the present Research Topic has been designed to demonstrate the current level of research and progress in the study of molecular, physiological, and genetic responses and adaptation strategies toward multiple abiotic stresses in cereals. The insights of this Research Topic are divided into the following headings:

## 1 Genetic studies and genome-wide association mapping

Characterization and identification of the differential responses of crop plants are one of the major essential steps for the development of climate-resilient crop plants. Amro et al. studied the growth responses and genetic variation among wheat genotypes for salinity tolerance (seawater) and identified a high genetic diversity among the studied genotypes, which could be utilized for breeding programs. Likewise, Radha et al. reviewed the individual and interactive effects of various abiotic stresses (drought, salinity, high temperature, eCO2, submergence, nutrient deficiency) and their combined effect on rice physiology with the possible adaptation strategies for improving grain quality parameters and yield traits. With the advancement of various tools in system biology (high-throughput phenotyping and genome sequencing), different approaches have been developed, such as quantitative trait locus (QTL) mapping, candidate gene association studies, and genome-wide association studies (GWAS), in order to link phenotypes and genotypes in crop plants for the identification of genetic factors associated with the various traits under consideration (Mir et al., 2019). Among them, GWAS is one of the most powerful tools for investigating complex traits associated with single or multiple abiotic stresses. It detects marker–trait association (MTA) using conserved linkage disequilibrium (LD) present in the selected panel of accessions (Myles et al., 2009; Saini et al., 2022). GWAS has a high capacity to identify small-effect genes/MTAs on a genome-wide scale by efficiently using the multiple historical crossover events that occur in the diverse association panel used (Saini et al., 2022; Xiao et al., 2022). In the present Research Topic, Devate et al. used a 35k SNP wheat breeder’s genotyping array to identify 57 unique markers associated with various traits across the locations for drought and heat tolerance in wheat. Out of these associations, 23 MTAs were deemed to be stable. Similarly, in another study, Devate et al. identified six unique marker–trait associations for grain iron (GFeC), zinc (GZnC) contents, and thousand-grain weight (TGW) under drought and heat stress conditions in wheat. These identified MTAs could be utilized in the breeding program after validation through marker-assisted selection (MAS).

## 2 Marker-assisted selection and abiotic stresses

The trait-specific markers (linked markers) allow the efficient introgression of targeted genomic loci from the donor genotype into an elite breeding line, facilitate indirect selection for difficult traits (i.e., root traits under drought stress conditions), and cut the number of genes/QTL into a single genotype using the marker-assisted selection approach (Pandurangan et al., 2022; Rai and Pandey-Rai, 2021). This method is very rapid and cost-effective for genetic improvement after the identification of tightly linked markers associated with the trait under consideration. It is effectively implemented for the improvement of multiple abiotic stress tolerance in various crops, i.e., rice (heat tolerance (Lang et al., 2015); submergence and drought tolerance (Kumar et al., 2020); drought, salinity, and submergence (Muthu et al., 2020); drought and heat stress (Withanawasam et al., 2022), wheat (drought tolerance (Ciucă et al., 2009; Merchuk-Ovnat et al., 2016; Rai et al., 2018; Gautam et al., 2021), and maize (drought (Ribaut and Ragot, 2007)). Similarly, in the present Research Topic, Sunilkumar et al. introgressed the rust resistance gene Lr24 and QTLs linked to moisture deficit stress tolerance in the background of HD3086 (a high-yielding, stress-susceptible genotype of wheat) from the HI1500 donor genotype.

## 3 Omics-based approaches and transgenics for multiple abiotic stresses

Understanding differential levels of plant mechanisms under multiple stress conditions is essential for combating their effect. Adopting different omics approaches (genomics, transcriptomics, proteomics, and metabolomics) and understanding their overall phenotypic effects on crop plants under abiotic stress conditions are crucial to developing strategies for designing crops with superior tolerance mechanisms (Bhardwaj et al., 2021; Jeyasri et al., 2021). Among the different omics approaches, transcription factors (TFs) are crucial for recognizing the appropriate molecular processes and pathways under stress conditions (Muthuramalingam et al., 2018; Muthuramalingam et al., 2020). In the present Research Topic, Annum et al. studied a phospholipase C (PLC) signaling pathway in spring wheat and evaluated its four AtPLC5 overexpressed (OE)/transgenic lines under heat and osmotic stresses through ^32^Pi radioactive labeling. The results indicate that heat stress and osmotic stress activate several lipid responses in wild-type and transgenic wheat conforming to osmotic stress tolerance. Kumar et al. studied the differential transcript expression of K+ transport genes in different tissues (root, stem, and leaf) under different abiotic stresses, such as salt, drought, heat, and cold, to elucidate their role in ion homeostasis and stress tolerance mechanisms in sorghum. Maheshwari et al. reviewed paclobutrazol (PBZ) as a plant growth regulator and multistress protectant; they discussed current findings and the prospective application of PBZ in regulating crop growth and ameliorating abiotic stresses. In another study, Kumar et al. used the 20S proteasome gene family in rapeseed and identified 20S proteasome genes for α- (PA) and β-subunits (PB) through systematically performed gene structure analysis. Out of 82 BnPA/PB genes, three exhibited high expression under abiotic stresses. Likewise, Ahmed et al. proposed a novel activation function, the Gaussian Error Linear Unit with Sigmoid (SIELU), for the deep learning (DL) model along with other hyperparameters for the classification of unknown abiotic stress protein sequences from cereal crops.

The papers presented in the current Research Topic are associated with the multiple abiotic stresses on various crops and show wider scope to understand the molecular, physiological, and genetic responses of multiple abiotic stresses. At the same time, the findings of the papers presented show a wide range of advanced scientific approaches and research ideas to understand and identify the effects of multiple abiotic stress and the implementation of their adaptation strategies for the development of climate-resilient crop plants.

## Author contributions

NB wrote the draft editorial. All authors edited it. All authors contributed to the article and approved the submitted version.
